# Surface passivation and optical characterization of Al_2_O_3_/a-SiC_x_ stacks on c-Si substrates

**DOI:** 10.3762/bjnano.4.82

**Published:** 2013-11-06

**Authors:** Gema López, Pablo R Ortega, Cristóbal Voz, Isidro Martín, Mónica Colina, Anna B Morales, Albert Orpella, Ramón Alcubilla

**Affiliations:** 1 Electronic Engineering Department, Polytechnic University of Catalonia, Jordi Girona 1-3, Mòdul C4, 08034 Barcelona, Spain

**Keywords:** aluminum oxide (Al_2_O_3_), antireflection coating, atomic layer deposition, silicon carbide (SiC_x_), surface passivation

## Abstract

The aim of this work is to study the surface passivation of aluminum oxide/amorphous silicon carbide (Al_2_O_3_/a-SiC_x_) stacks on both p-type and n-type crystalline silicon (c-Si) substrates as well as the optical characterization of these stacks. Al_2_O_3_ films of different thicknesses were deposited by thermal atomic layer deposition (ALD) at 200 °C and were complemented with a layer of a-SiC_x_ deposited by plasma-enhanced chemical vapor deposition (PECVD) to form anti-reflection coating (ARC) stacks with a total thickness of 75 nm. A comparative study has been carried out on polished and randomly textured wafers. We have experimentally determined the optimum thickness of the stack for photovoltaic applications by minimizing the reflection losses over a wide wavelength range (300–1200 nm) without compromising the outstanding passivation properties of the Al_2_O_3_ films. The upper limit of the surface recombination velocity (*S*_eff,max_) was evaluated at a carrier injection level corresponding to 1-sun illumination, which led to values below 10 cm/s. Reflectance values below 2% were measured on textured samples over the wavelength range of 450–1000 nm.

## Introduction

Surface passivation has become a relevant issue in high efficiency crystalline silicon (c-Si) solar cells. The importance is even increasing as thinner wafers are used to reduce the cost for photovoltaic applications [1]. Aluminum oxide (Al_2_O_3_) grown by atomic layer deposition (ALD) is a good alternative for passivating both lightly and highly doped n*-* and also p-type c-Si substrates [2–4]. The excellent passivation quality is due to a double effect: (i) chemical passivation that involves a low density of interface defects, *D*_it_ (≈10^11^ eV^−1^cm^−2^), and (ii) field-effect passivation due to a high negative fixed-charge density, *Q*_fix_ (≈10^12^ cm^−2^) [5–8], which acts as an electrostatic shielding and significantly reduces the density of one type of charge carrier at the interface c-Si/Al_2_O_3_ [9–10]. In order to achieve the lowest surface recombination velocity (*S*_eff_), it is necessary to perfom a thermal treatment after deposition (post-deposition annealing) to activate the passivating properties of Al_2_O_3_ layers [11–12]. In a previous work [13] we showed that an annealing process for 10 to 20 min at temperatures between 350 °C and 400 °C is enough to obtain an excellent passivation on polished p*-*type c-Si substrates.

In this work, we complement our preceding work by studying the surface recombination velocity on both n*-* and p*-*type wafers (polished and randomly textured), which were passivated with Al_2_O_3_/a-SiC_x_ stacks. In previous works we demonstrated that an a-SiC_x_ capping layer on the Al_2_O_3_ improves the laser contact formation on p-type c-Si solar cells in comparison to the typical laser fired contact (LFC) process [14–15]. Moreover, it is well known that the ALD deposition of Al_2_O_3_ has very low deposition rates. Inserting an a-SiC_x_ capping layer by PECVD technique can overcome this drawback. In this study, we have investigated different combinations of layers that provide good antireflection properties while maintaining a total film thickness of 75 nm. In addition to the passivation, a high-quality antireflection coating (ARC) plays a vital role in highly efficient solar cells [16]. We have measured the reflectance over a wide wavelength range, 300–1200 nm, in order to determine the optimum layer thicknesses for the stack to be used as an ARC without compromising the surface passivation quality.

## Results and Discussion

### Surface recombination results

**Figure 1:**
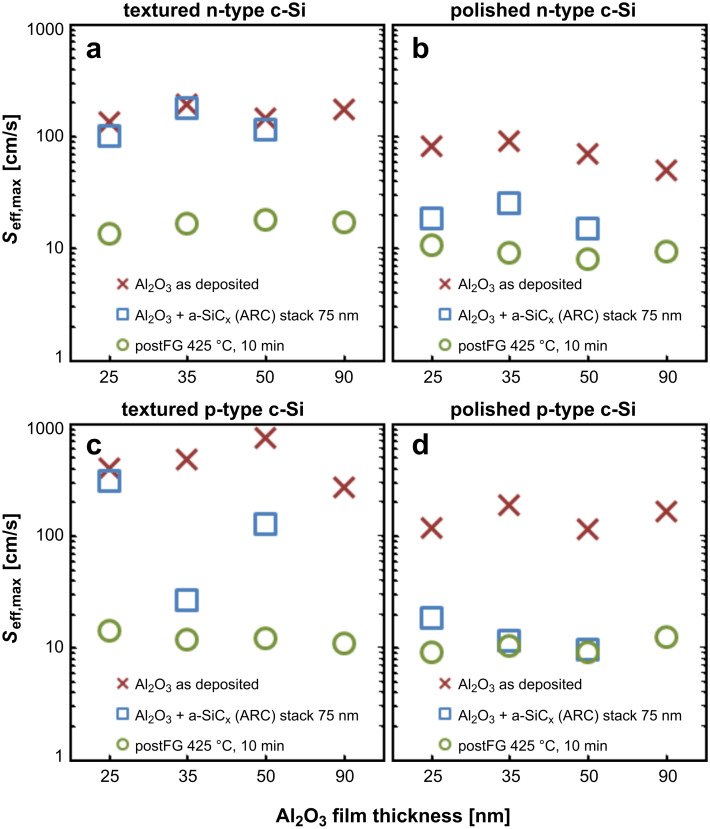
Surface recombination velocity, *S*_eff,max_ [cm/s]. **(a)** and **(b)** *S*_eff,max_ for randomly textured and polished n-type wafers respectively. **(c)** and **(d)** *S*_eff,max_ for randomly textured and polished p-type wafers respectively. *S*_eff,max_ was determined at 1-sun injection level as a function of the Al_2_O_3_ thickness. The aluminum oxide layers were complemented up to 75 nm with an a-SiC_x_ film.

The passivation characteristics of the c-Si/Al_2_O_3_/a-SiC_x_ stacks were tested the deposition of Al_2_O_3_ and a-SiC_x_, and after a final post-deposition annealing process (Figure 1). Moderate *S*_eff,max_ values were achieved for as-deposited Al_2_O_3_ layers with better results on polished than on randomly textured samples. This level of surface passivation can be explained by the relatively low *D*_it_ (≈10^11^ eV^−1^cm^−2^) prior to the annealing step [17–18], which is responsible for the chemical passivation. The higher *S*_eff,max_ results obtained on textured samples (>130 cm/s) could be attributed to a higher surface area due to the pyramid-shaped surface and a higher *D*_it_ value on the exposed {111} planes [19–21]. Regarding the field-effect passivation, it has been reported that ALD Al_2_O_3_ films exhibit a low *Q*_fix_ present at the c-Si/Al_2_O_3_ interface (≈10^11^ cm^−2^) prior to the annealing step [18]. Under these conditions, the electrostatic shielding of the interface does not induce an increase of the τ_eff_ value and the effect of the chemical passivation becomes more determinant. After the a-SiC_x_ deposition by PECVD, we observe in general a considerable improvement of the *S*_eff,max_ parameter. Values close to 10 cm/s were achieved on polished n- and p-type samples. This effect can be attributed to a small in-situ annealing effect that takes place in the PECVD chamber (deposition temperature *T*_dep_= 300 °C). The improvement of the passivation quality after an annealing step has been widely reported [3,7,18,22], and it has been related to a lower *D*_it_ ≤ 1 × 10^11^ eV^−1^cm^−2^ [17] combined with a higher concentration of fixed negative charges. The presence of these charges provides an electrostatic shielding due to a built-in electric field at the c-Si/Al_2_O_3_ interface [4,23]. Here, we also see that textured substrates showed higher *S*_eff_ values after the SiC_x_ deposition.

The final annealing treatment at *T*_ann_ = 425 °C for 10 min in forming gas improved the surface passivation, which led to a significant decrease in *S*_eff,max_ for most of the samples. As a result, outstanding *S*_eff,max_ values of less than 10 cm/s, i.e., τ_eff_(1 sun) > 1.3 ms, were achieved independently of doping and surface morphology. We have to note that the final values on randomly textured substrates were quite similar to those of polished ones. Thus, the annealing temperature is a crucial parameter to activate the surface passivation and it should be higher for textured samples (*T*_ann_ = 425 °C) than that found for polished substrates in a previous work (*T*_ann_ = 375 °C) [13]. In fact, some polished samples already showed an optimum passivation quality just after the PECVD process. On the other hand, an annealing temperature of 425 °C is a critical limit to avoid a blistering effect, which we have observed on polished samples with a 90 nm thick Al_2_O_3_ layer. The blistering effect consists in a partial delamination of the Al_2_O_3_ film and the corresponding bubble formation. It is caused by a gaseous desorption where the layer acts as a gas barrier. The density and dimensions of the bubbles are directly related to the annealing and the ALD process temperatures and the thickness of the Al_2_O_3_ layer [13,24–25].

Regarding the effect of the film thickness, it is interesting to note that a rather constant high level of surface passivation is obtained after the annealing for the whole range of Al_2_O_3_ thicknesses. The field-effect passivation remains constant independently of the thickness of the alumina layer probably because fixed negative charges seem to be located at the interface between Al_2_O_3_ and c-Si [6]. Other authors have demonstrated that a thin interfacial SiO_x_ layer between the c-Si and the Al_2_O_3_ film and generated during the Al_2_O_3_ deposition process, plays an important role in the formation of the negative fixed-charge density [26–30].

### Optical properties of Al_2_O_3_ and the Al_2_O_3_/a-SiC_x_ stack

The refractive index of Al_2_O_3_ measured by ellipsometry was around 1.6 at a wavelength of 632 nm, whereas for the a-SiC_x_ layers it was quite close to 2.0. On the other hand, the absorbance of Al_2_O_3_/a-SiC_x_ stacks deposited on borosilicate glass was analyzed by means of an UV–vis–NIR Spectrometer equipped with an integrating sphere in the wavelength range from 300 to 600 nm. The stack absorbance was calculated from the reflectance and transmittance measurements following Equation 1 and Equation 2,

[1]
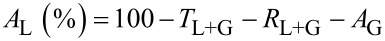


**Figure 2:**
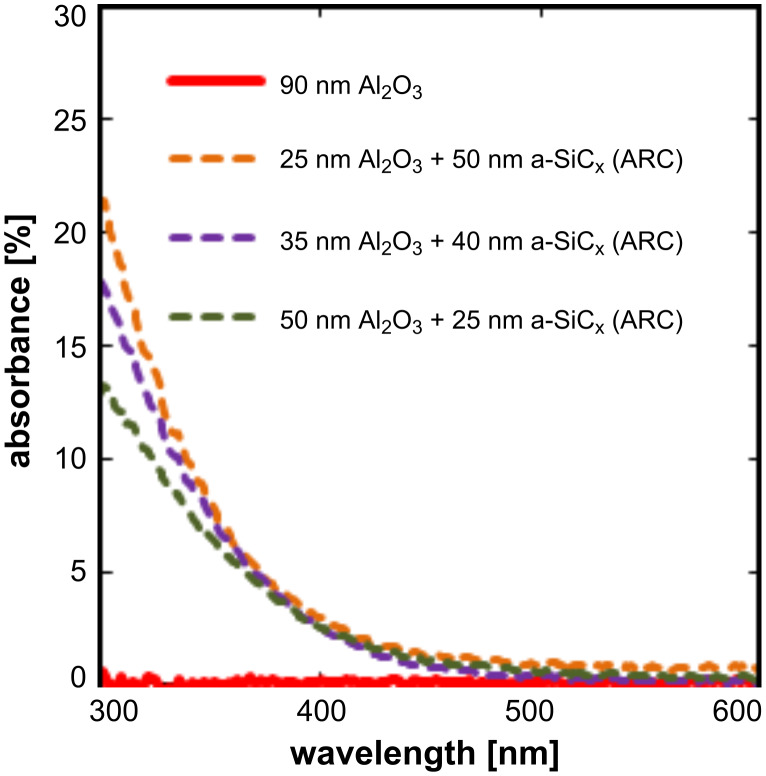
Absorbance as a function of the wavelength in the range from 300 to 600 nm. Dashed lines belong to stacks with different Al_2_O_3_ thicknesses while the continuous red line is the absorbance of 90 nm Al_2_O_3_.

[2]
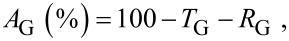


where *A* is the absorbance, *T* the transmittance and *R* the reflectance. The subscritps L and G correspond to the layer stack and glass respectively. The results of the absorbance measurements are shown in Figure 2.

Other works have previously reported an optical bandgap of *E*_opt_ = 6.4 ± 0.1 eV for as-deposited and annealed ALD Al_2_O_3_ films [18]. This means that this material is transparent for wavelengths above 200 nm. Therefore, absorption of light by the Al_2_O_3_ layer does not occur in the wavelength range relevant for photovoltaic applications. For the sake of clarity, only the 300 to 600 nm wavelength range is depicted, i.e, in which a relevant absorbance can exist. However, it can be seen that as the a-SiC_x_ layer thickness increases, the optical absorbance also increases up to a value of 21.1% at 300 nm. Thus, the SiC_x_ capping layer is less attractive to be used as an antireflection layer on the illuminated side of the solar cell compared to a single 90 nm Al_2_O_3_ film.

**Figure 3:**
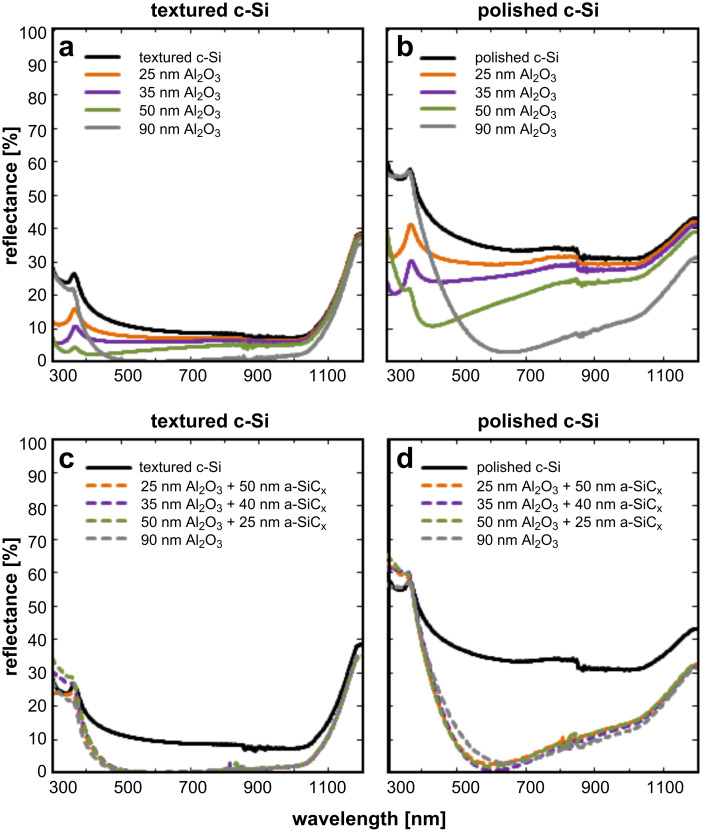
Reflectance curves of Al_2_O_3_-coated randomly textured c-Si **(a)** and polished c-Si **(b)** for different film thicknesses. Reflectance curves of Al_2_O_3_/a-SiC_x_ coated randomly textured c-Si **(c)** and polished c-Si **(d)**. As a reference, bare polished and textured c-Si reflectances are also included (black line).

Reflectance measurements were also carried out. No significant differences were found between n and p-type c-Si substrates. These measurements of single Al_2_O_3_ films as well as Al_2_O_3_/SiC_x_ stacks are shown in Figure 3.

The textured sample without ARC coating (black lines in Figure 3, left) exhibits an integrated average reflectance of 13.3%, much lower than that of polished c-Si substrates. For a randomly textured surface, the reduced reflectance is explained by a second reflection of the incident light at the sidewalls of an opposite pyramid [31]. After coating the silicon substrates, the optical reflectance was further reduced.

When a 90 nm Al_2_O_3_ film is deposited on a polished surface, the reflectance yields a minimum of 3.2% at a wavelength of about 600 nm, but it increases quickly for shorter and longer wavelengths. Moreover, the reflectance properties become worse as the Al_2_O_3_ film thickness decreases. Next, the reflectance is strongly reduced when the polished samples are coated, but it still increases for longer wavelengths.

On the other hand, when a textured c-Si sample is coated by 90 nm of Al_2_O_3_ the reflectance values measured are below 2% between 460 and 1000 nm. Similar results were obtained with the textured samples coated by 75 nm Al_2_O_3_/a-SiC_x_ stacks. Thus, both 90 nm Al_2_O_3_ film and 75 nm Al_2_O_3_/a-SiC_x_ stacks on textured surfaces are excellent anti-reflection options.

## Conclusion

Al_2_O_3_ layers and Al_2_O_3_/a-SiC_x_ stacks with different thicknesses were deposited on polished and randomly textured p*-* and n*-*type c-Si substrates by combining thermal ALD and PECVD technique. Outstanding *S*_eff,max_ values below about 15 cm/s were achieved independently of the surface morphology and doping type of the samples. This value is low enough to obtain highly efficient c-Si solar cells. Concerning the optical properties, the absorbance of Al_2_O_3_ layers with different thicknesses and also of different Al_2_O_3_/a-SiC_x_ stacks was calculated by evaluating reflectance and transmittance measurements. We found that the absorption loss in a-SiC_x_ layers in the range of short wavelengths of is the reason for the superior overall optical performance of a single 90 nm thick Al_2_O_3_ film. Therefore, the latter represents the better option as an antireflection coating compared to Al_2_O_3_/a-SiC_x_ stacks. This result is supported by the reflectance measurements of Al_2_O_3_ films with different thicknesses and Al_2_O_3_/a-SiC_x_ stacks on polished and textured c-Si substrates in the wavelength range from 300 to 1200 nm. In any case, the measured reflectance was less than 2% for all the Al_2_O_3_/a-SiC_x_ stacks and also for the single 90 nm layer of Al_2_O_3_. Nevertheless, an a-SiC_x_ capping layer could be useful if the Al_2_O_3_ layer needs to be protected from some chemical treatment during the solar cell fabrication. Moreover, on the rear side of a c-Si solar cell, where the optical absorbance is not critical, an a-SiC_x_ layer on top of the passivated Al_2_O_3_ film acts as a back reflector that reflects photons towards the bulk. This a-SiC_x_ capping layer on the Al_2_O_3_ also improves the laser contact formation on p-type c-Si solar cells.

In summary, we can conclude that a 90 nm Al_2_O_3_ film on textured c-Si substrates results in a good scheme for both passivation and anti-reflection coating on the illuminated side of highly efficient solar cells, whereas an a-SiC_x_ capping layer on Al_2_O_3_ films on the rear side of the solar cell provides better back contacts and a better back reflector scheme.

## Experimental

As starting material, n*-* and p*-*type (2.5 ± 0.3 Ωcm) FZ silicon(100) wafers with a thickness of approximately 290 µm were used. One p*-*type and one n*-*type wafer were textured on both sides with solution of tetramethylammonium hydroxide (TMAH) in isopropanol (IPA) solution. Before film deposition, four wafers, two n*-*type (one textured and one polished) and two p*-*type (one textured and one polished) were cleaned following an RCA sequence and cut to quarters. Al_2_O_3_ films were subsequently deposited by thermal ALD (Savannah S200, Cambridge Nanotech; Cambridge, MA, USA) at *T*_dep_ = 200 °C. This technique is based on sequential, self-limiting chemical reactions at the surface. The surface of the substrate is exposed to the precursor gases in alternating manner. The reactions are cyclical and after each reaction, there is a purge with N_2._ The typical ALD cycle to deposit Al_2_O_3_ layers consists of the injection into the chamber of trimethylaluminum (Al(CH_3_)_3_) for 15 ms followed by N_2_ purging and the injection of water vapour for 50 ms followed by N_2_ purging. The precursor doses and exposure times were chosen such that all films were deposited under saturated self-limiting conditions leading to a film growth of 1.1 Å/cycle. On each sample, belonging to a different type of wafer, films with a thickness of 25, 35, 50 and 90 nm, respectively, were deposited (deposition times of 38, 53, 73 and 137 min respectively). On top of these films, we deposited an amorphous silicon carbide (a-SiC_x_) film by PECVD that uses silane (SiH_4_) and methane (CH_4_) as precursor gases. The thicknesses of these a-SiC_x_ films were 50, 40 and 25 nm, respectively (deposition times of 12 min 50 sec, 10 min 15 sec and 6 min 24 sec, respectively) in order to complement the 25, 35 and 50 nm Al_2_O_3_ films for a total stack thickness of 75 nm. Substrates with an Al_2_O_3_ thickness of 90 nm were also studied without any a-SiC_x_ capping layer. A post-deposition annealing process in a forming gas environment (H_2_/N_2_) at 425 °C for 10 min was done to activate the passivation properties. All these substrates were symmetrically covered to measure the effective lifetime τ_eff_ by measuring the quasi-steady-state photoconductance (QSSPC) with a Wafer Lifetime Tester Sinton WCT-100 [32–33]. The upper limit of the surface recombination velocity (*S*_eff,max_) was deduced from the effective lifetime τ_eff_ measurements as a function of the excess carrier density (Δ*n*) at 1 sun injection level as

[3]
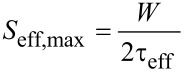


where *W* is the substrate thickness and an infinite bulk lifetime has been assumed.

Concerning the optical characterization, the thickness and refractive index of the individual Al_2_O_3_ and SiC_x_ layers were measured by ellipsometry (Plasmos SD 2100) at a wavelength of 632 nm. Finally, in order to know the optical absorbance, we also deposited the same Al_2_O_3_/a-SiC_x_ stacks on transparent substrates (Borosilicate glass). The reflectance (diffuse and specular) and the transmittance were measured in the wavelength range from 300 to 1200 nm by using a UV–visible–NIR spectrometer (Shimadzu 3600) equipped with an ISR 3100 integrating sphere.
